# Supplementary material for: Measurement in the study of menstrual health and hygiene: A systematic review and audit
Source: PLoS One. 2020 Jun 4;15(6):e0232935. doi: 10.1371/journal.pone.0232935 (PMC7272008; doi:10.1371/journal.pone.0232935)
Supplement: S2 Table — (PDF) [file pone.0232935.s002.pdf]

Supplementary Materials 2. Author reported concepts measured in trials and nested studies, assessment modality and development

Trials<br>Study ID	Trial outcomes (O) and descriptive measures (D)*	Assessment method	Measure development or validation
Abedian 2011[12]	(O) Menstrual cycle characteristics (pain and symptoms)<br>(O) Menstrual knowledge<br><br>(O) Menstrual attitudes	Self-report written survey	Designed by investigators, and criteria from DSM-IV-TR premenstrual syndrome<br>Designed by investigators based on textbooks.<br>Menstrual Attitude Questionnaire
Beksinska 2015[13]	(O) Acceptability and performance of products<br><br>(O) Adverse events<br>(D) Household WASH<br><br>(D) Menstrual practices and experience	Self-report questionnaire (format NR)<br>Product use diary<br>Self-report<br>Self-report questionnaire (format NR)	NR<br><br><br>NR<br>NR<br><br>NR
Blake 2018[14]	(O) Menstrual and puberty knowledge<br><br>(O) Attitudes towards menstruation<br>(O) Menstrual fear and shame<br>(D) Menstrual practices<br>(D) WASH observations	Self-report written survey with verbal description in class<br><br><br><br><br>Observational checklist	Adapted from existing evaluations of puberty education materials.<br><br>Field tested with girls in other schools.
Dhanalakshmi 2015[15]	(O) Menstrual/puberty knowledge<br>(O) Beliefs about menstruation<br>(O) Menstrual practices (and sexual behaviours)<br>(D) Menstrual symptoms	Self-administered [NR]	NR
Djalalinia 2012[16]	(O) Menarche experience<br><br>(O) Menstrual information (source acceptability)<br>(O) Menstrual and hygiene practices<br>(D) Menstrual pain and symptoms	Self-report written survey	Designed by scientific committee and sent to 10 experts. Stated reliability but not reported.
El-Mowafy 2014[17]	(O) Knowledge<br><br>(O) Self-care practices (including menstrual practices)	Self-report in interview	Developed by investigators. Reviewed by 3 experts. Piloted on 10% of sample
Fakhri 2012[18]	(O) Menstrual health	Self-report in interview	Developed by investigators, tested via input from adolescents, scientists and community experts. Test-retest reliability with 20 students.
Fetohy 2007[19]	(O) Menstrual knowledge<br><br>(O) Menstrual attitudes	Self-report written survey	Designed by investigators, pilot-testing stated with no further detail.

	(O) Menstrual behaviour		
Leventhal 2016[20]	(O) Overarching trial outcomes included general health knowledge, gender equality attitudes, WASH, hand washing and health care utilisation<br>(O) Psychosocial wellbeing, resilience, social-emotional assets<br>(O) Menstrual hygiene	Self-report written surveys in groups with assistants to help with reading/understanding	Pre-tested questionnaires including investigator-developed and previously established measures. Piloted with 74 girls in neighbouring area.
Mbizvo 1997[21 22]	(O) Reproductive health and menstrual knowledge	Self-report written survey	NR
Mohamed 2014[23]	(O) Discomfort/comfort with products after physical activity<br>(O) Physical exertion	Self-report on visual analogy scale and survey.<br>Heart rate variability measured using electrocardiograph.<br>Self-reported exertion.<br>Heart rate low-frequency to high-frequency ratio.	NR<br><br>Previously validated measures/ biomarkers.
Montgomery 2012[24 25]	(O) School attendance	Attendance registers	Attendance registers were compared with spot-checks and found to be reliable.
	(D) Menstrual cycle and product use	Self-completed menstrual diaries	NR
	(O) Subjective shame and well-being	Self-report with interviewer	Adapted from established/ recommended dimensions of noneconomic poverty.
	(O) Classroom participation	Self-report with interviewer	NR
Montgomery 2016[26]	(O) School attendance	Attendance registers/ spot-checks	
	(O) Wellbeing	Self-report with interviewer (responses entered electronically)	Tools piloted in Ghana during pilot (Montgomery 2012)
	(O) Subjective shame<br>(O) Menstrual knowledge		
	(D) WASH facilities	Observational checklist	NR
Oster 2011[29]	(O) School attendance (during menstruation)	School registers combined with self-completed menstrual diaries	NR.
Phillips-Howard 2016[31]	(O) School attendance	Self-completed calendars	
	(O) School drop out	Absence from nurse visits	NR
	(O) Sexually transmitted or reproductive tract infection	Self-taken vaginal swabs.	Lab testing for presence of STI/RTI
	(O) Adverse events	TSS - vaginal swabs	
	(D) Menstrual characteristics	Self-report survey. NR further details	NR
	(D) Wellbeing	Self-report survey.	Paediatric Quality of Life Inventory (PEDSQL)
	(D) WASH facilities	Observation checklist	NR
Sener 2019[37]	(O) Hygiene skills and behaviours (including menstrual hygiene)	Self-report in interview with study staff + study staff repeated observations.	Researcher developed based on past literature with review by 10 experts. Piloted on 5 non-participating teens and parents.

Setyowati 2019	(O) Knowledge about menstruating and puberty<br>(O) Emotional responses to menarche<br>(O) Menstrual attitude questionnaire	NR.	Developed in past research and adapted for context.<br>Questions developed from past studies<br>Previously validated measure: Menstrual Attitude Questionnaire
Sharma 2015[39]	(O) Menstrual knowledge<br><br><br><br>(O) Menstrual hygiene practices	Survey. NR.	Researcher developed questionnaire with input from expert. Post-hoc reliability calculated.
Shrestha 2018[40]	(O) Demand for menstrual products<br><br>(D) Menstrual knowledge<br><br>(D) Menstrual stigma and norms<br>(D) Health and sanitation (incl. Menstrual practices)	Coupon redemption in local stores.<br><br>Self-report with interviewer	Researcher spot checks on store-recorded coupon redemption and physical storage of coupons.<br>NR
Stadler 2006[41]	(O) Physical discomfort<br><br>(O) Product acceptance<br>(D) Product use and menstrual practices	Self-reported questionnaire<br><br>Product use diary (self-completed)	NR<br><br><br>NR
Valizadeh 2017 [42 43]	(O) Menstrual knowledge<br><br><br>(O) Menstrual practices<br>(O) Menstrual attitudes	Self-report survey (NR methodology)	Questionnaires adapted from past studies and checked by 11 faculty. Post-hoc reliability reported.
Wilson 2014[44 45]	(O) School attendance<br><br><br><br>(D) Acceptability of intervention<br>(D) Menstrual hygiene practices	Self-reported written survey with facilitator providing verbal translation	NR
Womena 2018[46]	(D) Menstrual practices<br><br>(D) Menstrual factors<br>(D) Menstrual product preference and experience<br>(D) Menstrual knowledge<br>(O) Experiences of the intervention/products	Self-reported in interview recorded electronically<br>Self-completed diaries<br>Surveys and diaries<br><br>Self-completed form test<br>Self-reported in interview recorded electronically	Pilot evaluation designed to test the acceptability of tools
**Nested Study ID**	**Variables measures as Predictors (P), Outcomes (O) or for Description (D)**	**Assessment method**	**Measure development or validation**
Hennegan 2016a[27]	(P) Menstrual practice<br><br><br>(O) Product preference and experiences<br>(O) Menstrual restrictions	Self-report with interviewer (responses entered electronically)	Tools piloted past study (Montgomery 2012)
Hennegan 2016b[28]	(P/O) Menstrual hygiene<br><br><br>(O) Health and symptoms	Self-report with interviewer (responses entered electronically)	Tools piloted past study (Montgomery 2012)

	(O) Education engagement<br>(O) Psychosocial wellbeing		
Oster 2012[30]	(P) Peer product use<br>(O) Menstrual cup use/adoption	Self-report survey<br>Interview with nurse<br>Monthly diaries	NR<br>NR
Nyothach 2015[32]	(O) Handwashing during menstrual practices<br>(P) Menstrual product used	Self-completed electronic survey	Piloted paper questionnaires prior to use in electronic netbooks
Odour 2015[33]	(O) Dropping menstrual items and disposal<br>(P) Menstrual product use	Self-completed electronic survey	Piloted paper questionnaires prior to use in electronic netbooks
Juma 2017[34]	(O) Adverse health outcomes	Self-collected vaginal swabs with lab testing	N/A
Van Eijk 2018[35]	(O) Observed menstrual cup colour change<br>(O) Observed menstrual cup damage<br>(P) Self-reported menstrual cup use<br>(P) Menstrual characteristics (time since menarche)	Nurse observation<br>Nurse observation<br>Self-reported interview with nurse<br>Self-completed electronic survey	NR<br><br>NR<br>Piloted paper questionnaires prior to use in electronic netbooks
Alexander 2018[36]	(O) Observed WASH facilities<br>(O) Teacher-reported WASH	Observational checklist<br>Teacher interview	 NR
Tolonen 2019	(O) School attendance<br>(D) Menstrual characteristics	School registers and spot checks<br>Self-report survey	Two methods compared. Spot checks were unannounced.<br>Piloted paper questionnaires prior to use in electronic netbooks

*Excludes demographic characteristics.
