# Supplementary material for: Measurement in the study of menstrual health and hygiene: A systematic review and audit
Source: PLoS One. 2020 Jun 4;15(6):e0232935. doi: 10.1371/journal.pone.0232935 (PMC7272008; doi:10.1371/journal.pone.0232935)
Supplement: S3 Table — (PDF) [file pone.0232935.s003.pdf]

Supplementary Materials 3. Review B: Development, reliability and validity of scale measures

Measure	Study	Development	Dimensionality	Reliability	Validity
**Attitudes, beliefs and stereotypes**					
Menstrual Attitude Questionnaire (MAQ) and<br><br>Menstrual Attitude Questionnaire – Adolescent Form (MAQ-A)	Brooks-Gunn 1980 (US)[55]	Theoretically driven by authors.	EFA using principal components analysis (varimax) in two consecutive samples. Broad replication of the factor structure was identified. Authors reported coefficients of congruence (0.77-0.91)	Cronbachs alpha (0.90-0.97 for all subscales)	Authors do not explicitly report validation.<br>Differences in sub-scale scores were compared between men and women, and between university women and adolescent pre and postmenarcheal girls.<br>Authors examined relationship between MAQ scores and self-reported menstrual symptoms through the menstrual distress questionnaire. The study compared symptomatology (e.g., pain, negative affect, concentration) to MAQ scores.
MAQ revalidation or testing	Bramwell 2002 (UK, India)[54]	NA	Dimensionality assessed in British and Indian samples using CFA. Structure was a poor fit for British (Comparative Fit Index (CFI)=0.52) and Indian sample (CFI=0.21). Authors also compared mean item ratings across samples.	NR	NR
	Firat 2009 (Turkish, Turkey)[59];	NA	CFA undertaken in high school (CFI=0.776, RMSEA=0.046) and university students (CFI=0.797, RMSEA=0.0527) in Turkey. Authors concluded poor fit based on CFI and significant Chi-square findings.<br>EFA using maximum likelihood and varimax rotation identified new factor structure, retaining items with loadings ≥0.40.	Cronbach's alpha for new scales ranged from 0.54 to 0.75.<br>Total score alphas were 0.73 for the high school and 0.79 for the university sample.	NR
	Bargoita 2016 (Greek, Greece)[53];	NA	CFA did not confirm the original factor structure. (χ^2=1136.9, $p<0.0005$, RMSEA=0.066, Goodness of Fit Index=0.755,	Cronbach's alpha for the original structure ranged from -0.150 to 0.778 (0.711 for the total)	NR

			CFI=0.784, AGFI=0.701, IFI=0.789, TLI=0.75, NFI=0.683).<br>EFA identified new factors and loadings ≥0.40 reported. Authors reported similar factors, but with some items changing sub-scales.	The new factor structure had Cronbach's alphas of 0.706-0.754 (0.711 for the total scale)	
	Stubbs 1988 (US)[71]	NA	Poor internal consistency of sub-scales were reported as poor support of existing factor structure.<br>Authors rescored items using a new strategy. They undertook an EFA using principal components analysis (no rotation)	Cronbach's alphas were found to be poor for subscales (0.05-0.61) Internal consistency for the new scales were α=0.69-0.62.	Concurrent validity was assessed through relationships with depressive and anxiety symptoms (Beck Depression Inventory, Children's Manifest Anxiety Scale), body satisfaction (Body Cathexis Scale), self-esteem (Rosenberg Self-Esteem Scale), and locus of control (Locus of Control Questionnaire).<br>In subsequent analyses authors used the scale to test the influence of age, menarche, and menarcheal timing on menstrual attitudes.
	Aubeeluck 2002 (UK)[52];	Assess priming affects of the MJQ based on author's position that existing scales stereotyped menstruation negatively.	NA	Authors explored order of presentation effects on MAQ scores. Finding that presentation of a positive 'Menstrual Joy Questionnaire' reduced negative reporting on one sub-scale.	
	Chrisler 1994 (US)[56]	Assess priming affects of the MJQ based on author's position that existing scales stereotyped menstruation negatively.	NA	Authors explored order of presentation effects on MAQ scores. Finding that presentation of a positive 'Menstrual Joy Questionnaire' increased reports of "menstruation as a natural event."	
The Stereotypic Beliefs About Menstruation Scale (SBAM)	Heard 1977 (US)[61]	Based on past literature of negative attitudes towards menstruation. Items developed from review of literature on myths, stereotypes and attitudes to menstruation.	Dimensionality was explored with EFA using principal components analysis.	Cronbach's alphas for sub-scales were 0.62,0.71,0.71,0.76	Authors do not explicitly report validation.<br>Differences in scores were compared between men and women.

Adolescent Menstrual Attitude Questionnaire (AMAQ)	Morse 1993 (Canada)[65 66]	Concepts and items developed through qualitative study.<br>Experts rated the relevance of items with poorly rated items removed.<br>Items were also piloted with a small number of girls.	EFA using principal components analysis with orthogonal varimax rotation resulted in the six-factor structure with some differences in item loading in structure apparent between those pre and post menarche.<br>No CFA was reported.	Internal consistency of subscales measured by Cronbach's alpha varied from α=0.62-0.90.	Discriminant analysis was undertaken to compare attitudes between girls pre- and post-menarche. Information was used to inform the scale.<br><br>Subscales were found to replicate dimensions identified through qualitative study.
Beliefs about and Attitudes Toward Menstruation Questionnaire (BATM)	Marvan 2006 (Mexico)[64]	Concepts were defined by authors, and in response to literature review.<br>Authors undertook consultation with women from different ages informally.<br>Experts rated items for face and content validity, with poor performing items removed.<br>Draft items were piloted in a small sample.	EFA using principal components analysis and varimax rotation was undertaken on the first sample to arrive at a five-factor structure.<br>A second data collection among North American and Mexican university students (n=274). Again, undertaking EFA through principal components analysis, an alternate four-factor solution was reached, although authors reported these factors were similar across both samples.	In the first sample internal consistency ranged from α=0.71-0.83)<br>In the second sample α=0.62-0.84)	Validity was assessed through the discriminant capacity of each item between highest and lowest scoring sub-groups.<br>Mexican and US samples were compared finding more behavioural proscriptions and prescriptions in the Mexican sample. Scores were compared between men and women.<br>Authors did not report any tests as tests of validity.
Menstrual Self-Evaluation Scale	Roberts 2004 (US)[68]	Developed to meet authors objectives of testing associations between menstrual self-evaluation and self-objectification.<br>Two sub-scales from the MAQ were used, with additional items generated by authors.	EFA through principal components analysis with varimax rotation was undertaken and arrived at four factors.<br>One original sub-scale from the MAQ was replicated.	Cronbach's alphas for the two subscales were 0.81 and 0.80.	As hypothesised, scores were correlated with age and with self-objectification.<br>Correlations of negative attitude scales with self-objectification but not the positive emotions factor.
**Health, hygiene, self-care and help-seeking**					
Female adolescents' sexual reproductive self-care scale	Alimoradi 2019 (Iran)[51]	Qualitative interviews were undertaken with adolescents to develop the concept and inform item development. Authors also reviewed past questionnaires.	EFA was undertaken to reduce items and identify factors. No CFA was reported.	Cronbach's alphas were 0.70-0.90 for subscales, and 0.895 for the total score. Test-retest among a subset of 20 students was 0.91 for the total (0.65-0.99 for subscales)	Face and content validity were assessed through feedback from adolescents and experts.<br>No further validation reported.
Menstrual health-seeking behaviors questionnaire (MHSBQ-47)	Darabi 2018 (Iran)[58]	Development informed by review of past research as well as qualitative research with adolescents.	EFA through principal components analysis with varimax rotation undertaken to explore factor structure. CFA assessed model fit for the six-factors (RMSEA=0.05, CFI=0.962, IFI=0.955, TLI=0.946, GFI=0.996, AGFI=0.995)	Cronbach's alphas were 0.92 for the overall scale and ranged from 0.79-0.91 for subscales.<br>Test-retest with 30 females at 2-weeks was 0.86 (total) and 0.91-0.94 for subscales.	Face and content validity was assessed by expert panel and feedback from adolescents.

Menstrual hygiene management	Khan 2017 (Belize)[63]	Questions were based on a definition of menstrual hygiene management facilities and consultation with experts.	NA	NA<br>Assessed the proportion of responses needing clarification.	Face and construct validity, as well as item performance assessed through cognitive interviews and focus group discussions with enumerators.<br>Interviewers needed to clarify or probe in 38-52% of interviews.<br>Questions were acceptable, terms "materials" and "privacy" caused some confusion.
Menstrual hygiene management scale (untitled)	Ramaiya 2019 (India)[67]	Development based on past framework, review of past research of menstrual hygiene management indexes, and expert committee. A qualitative study further informed interpretation.	EFA on baseline sample, using promax rotation Two factors were identified, these were not replicated in the endline sample. Endline EFA using principal components analysis found items loaded separately.	Not calculated due to poor factor loading.	Face and content validity rated by experts.<br>Qualitative study undertaken alongside aided interpretation of included items.<br>No further validation reported.
Menstrual Health Instrument	Shin 2018 (South Korea)[69]	Construct defined through review of past literature. Items reviewed by expert panel.	EFA using principal components and oblique rotation used to determine factors and shorten scale.	Cronbach's alpha was 0.62-0.94 for subscales. Test-retest at three weeks had ICCs from 0.46-0.88 for subscales.	Face and content validity assessed by experts and small number of adolescents.<br>Known groups validity was tested between those who had sought healthcare for menstrual problems and those who had not.<br>Criterion validity assessed through relationships between measure and menstrual symptoms, and quality of life measures.
**Impact of menstruation on school and wellbeing**					
Menstrual Related – School participation, stress and self-efficacy tool (MR-SSS)	Haver 2018 (Philippines; El Salvador)[60]	Concepts defined from existing theory (e.g., self-efficacy), and qualitative studies.	EFA and exploratory analyses undertaken but were inconclusive.	Authors report low interrater reliability measures.	NR

AGFI=Adjusted Goodness of Fit Index; CFA=Confirmatory Factor Analysis; CFI= comparative fit index; ECVI=Expected cross validation index; EFA=Exploratory Factor Analysis; GFI=Goodness of fit index; IFI=Incremental fit index; RMR=Root mean square residual; RMSEA=Root mean square error of approximation; TLI=Tucker-Lewis Index
